# Potential molecular mechanisms of Erlongjiaonang action in idiopathic sudden hearing loss: A network pharmacology and molecular docking analyses

**DOI:** 10.3389/fneur.2023.1121738

**Published:** 2023-03-30

**Authors:** He Zhao, Yan Wang, Cong Xu, Guangjin Li, Yuwan Song, Jingjing Qiu, Limei Cui, Xicheng Song, Yujuan Yang, Yan Sun

**Affiliations:** ^1^The Second Medical College, Binzhou Medical University, Yantai, Shandong, China; ^2^Department of Otolaryngology and Head and Neck Surgery, Yantai Yuhuangding Hospital, Qingdao University, Yantai, Shandong, China; ^3^Shandong Provincial Clinical Research Center for Otorhinolaryngologic Diseases, Yantai, Shandong, China; ^4^School of Clinical Medicine, Weifang Medical University, Weifang, China

**Keywords:** ISHL, TCM, Erlongjiaonang, bioinformatics, gene targets

## Abstract

**Background:**

Idiopathic sudden hearing loss (ISHL) is characterized by sudden unexplainable and unilateral hearing loss as a clinically emergent symptom. The use of the herb Erlongjiaonang (ELJN) in traditional Chinese medicine is known to effectively control and cure ISHL. This study explored the underlying molecular mechanisms using network pharmacology and molecular docking analyses.

**Method:**

The Traditional Chinese Medicine System Pharmacological database and the Swiss Target Prediction database were searched for the identification of ELJN constituents and potential gene targets, respectively, while ISHL-related gene abnormality was assessed using the Online Mendelian Inheritance in Man and Gene Card databases. The interaction of ELJN gene targets with ISHL genes was obtained after these databases were cross-screened, and a drug component–intersecting target network was constructed, and the gene ontology (GO) terms, Kyoto Encyclopedia of Genes and Genomes, and protein–protein interaction networks were analyzed. Cytoscape software tools were used to map the active components–crossover target–signaling pathway network and screened targets were then validated by establishing molecular docking with the corresponding components.

**Result:**

Erlongjiaonang contains 85 components and 250 corresponding gene targets, while ISHL has 714 disease-related targets, resulting in 66 cross-targets. The bioinformatical analyses revealed these 66 cross-targets, including isorhamnetin and formononetin on NOS3 expression, baicalein on AKT1 activity, and kaempferol and quercetin on NOS3 and AKT1 activity, as potential ELJN-induced anti-ISHL targets.

**Conclusion:**

This study uncovered potential ELJN gene targets and molecular signaling pathways in the control of ISHL, providing a molecular basis for further investigation of the anti-ISHL activity of ELJN.

## Introduction

Idiopathic sudden hearing loss (ISHL), a class of sudden sensorineural hearing loss (SSNHL), is characterized by sudden unexplainable and unilateral hearing loss. Clinically, ISHL can cause persistent tinnitus and/or hearing loss, leading to reduced quality of life. It affected an estimated 27 per 100,000 individuals in 2007 (1), with approximately 66,000 new cases identified annually in the USA (2). A Japanese study showed that the highest ISHL incidence rate occurred in patients aged 60 years or older, although the average age was 54 years old (3). To date, there are different approaches to SSNHL treatment, such as systemic and topical glucocorticoids, thrombolytic agents, antiviral agents, and drugs to improve microcirculation and nerve nourishment (4). Corticosteroids with or without combination with hyperbaric oxygen therapy (HBOT) may be used as initial therapy for the first 2 weeks, followed by combination therapy with HBOT as relief within the first month of disease (2). However, these treatments may not be able to cure all patients. For example, a recent study showed a cure rate of less than 30% after oral, intravenous, or tympanic administration of steroids in patients (5).

Traditional Chinese medicine, as one form of alternative medicine, utilizes botanical, mineral, or animal-derived agents to control or release human diseases and syndromes. According to Chinese Pharmacopoeia 2020, Erlongjiaonang (ELJN), as an oral capsule, is prescribed to treat dizziness, headache, deafness, tinnitus, and ear discharge as caused by the TCM theory of “damp heat” in the liver and gallbladder (6). A recent clinical trial of ELJN plus dexamethasone in the treatment of patients with SSNHL revealed that this regimen of treatment had better responses than the control group of dexamethasone alone (93.97% vs. 79.31%) (7). Another clinical trial data showed that ELJN plus gastrodin injection was more beneficial in SSHNL control than that of gastrodin injection alone (97.53% vs. 85.00%), and the adverse reactions were lower in the drug combination arm than in the control arm (6.17% vs. 20.00%) (8). However, to date, the underlying molecular mechanisms of ELJN in the treatment of ISHL remain unknown.

**Figure 1:**
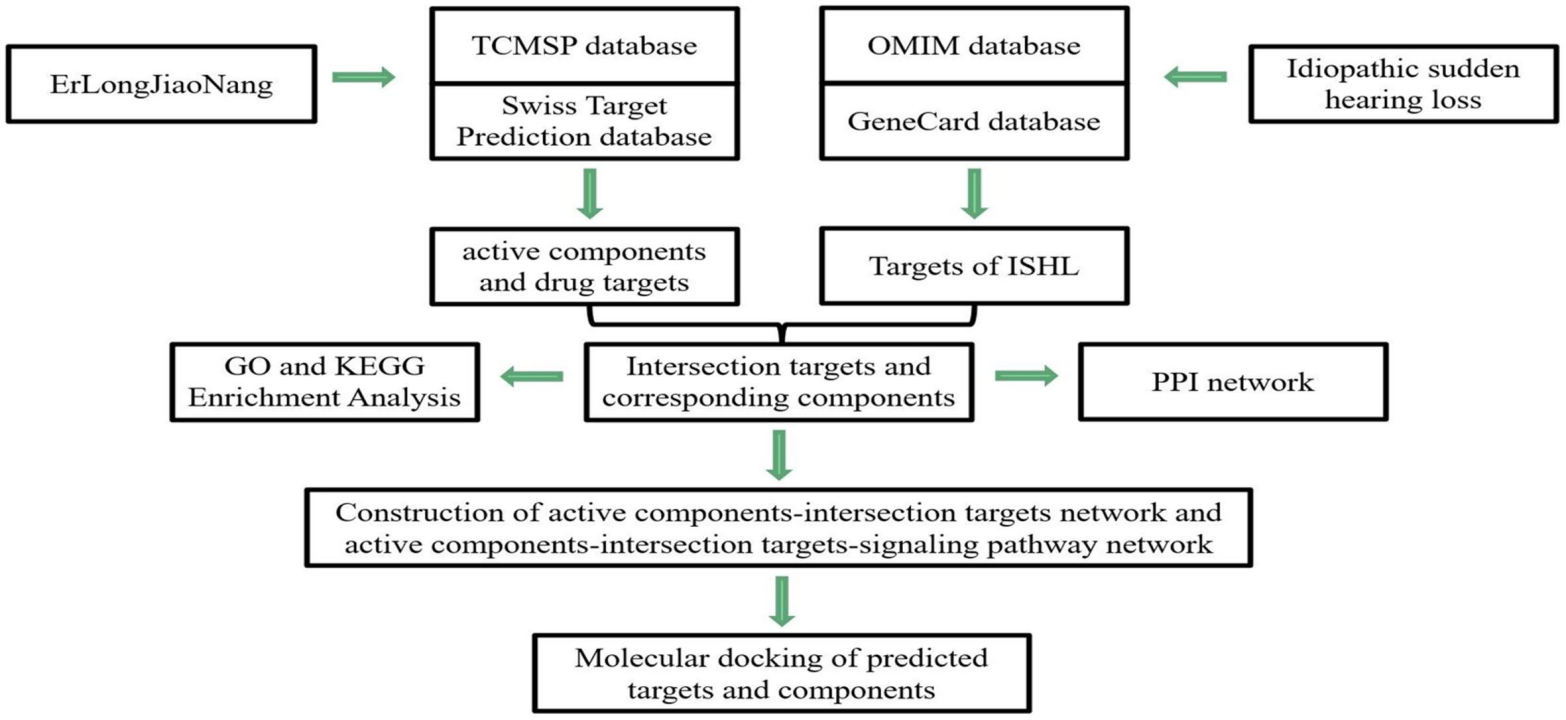
Illustration of study flow and data analysis. In this study, we first identified the possible active components in ELJN and then searched for their molecular targets. Meanwhile, we also identified ISHL-related genes. Subsequently, we constructed different networks to analyze the interaction of these two types of genes molecularly and then molecular docking analysis of the genes targeted by the active components of ELJN.

Network pharmacology refers to a combination of pharmacology with bioinformatics and systems biology to assess the multi-compound, multi-targets, and multi-pathway characteristics in TCM (9). Thus, in this study, we applied this novel analytic tool to construct the network of “effective components-action target” (drug–target network) and overlay the targets between ELJN and SSHNL (the drug–disease network), in order to discover the potential ELJN molecular targets on SSHNL. We analyzed different nodes after establishing networks to better understand the ELJN anti-SSHNL activity, which could provide us with a novel strategy for future SSHNL prevention and treatment and better understand SSHNL pathogenesis clinically (Figure 1).

## Materials and methods

### Screening of ELJN active components and therapeutic targets

Erlongjiaonang contains 10 different TCM herbs: Gardeniae Fructus, Alisma, Caulis Akebiae, Gentianae Radix Et Rhozima, Anemone Altaica Fisch, Rehmanniae Radix Praeparata, Angelicae Sinensis Radix, Licorice, Scutellariae Radix, and Antelope Horn. These herbs were used to search the 2020 Chinese Pharmacopoeia and the Traditional Chinese Medicine System Pharmacological (TCMSP) databases^1^ (10) to identify their constituents. The screening criteria used were: >30% oral bioavailability (OB) and > 0.18 drug-likeness (DL) (11), according to the TCMSP database. The chemical structure of each component was obtained from the chemical source network database, and the corresponding components were then mapped according to their chemical structures using the Swiss Target Prediction database^2^ for the species “Homo sapiens” (12) and the targets of each component were further screened. Subsequently, the validated human targets were then screened using the Uniprot database^3^ (13), and the targets from these two databases were combined, while the target names (symbols) were corrected to match the official names in this database.

### Screening of ISHL therapeutic targets

The terms “idiopathic sudden deafness” and “idiopathic sudden hearing loss” were used as the keywords when searching the Online Mendelian Inheritance in Man (OMIM) database^4^ (14, 15), Gene Cards database^5^ (16), and the Therapeutic Target Database (TTD)^6^ (17). The resulting data were collected from each database and summarized in an *Excel* file.

### Construction of the component–network–pathway network

We mapped the potential ELJN gene targets and ISHL targets with a Venn diagram using the Venny2.1 tool^7^ and Cytoscape software (version 3.9.1) to create an ELJN active components–intersection target–signaling pathway network map using the data from the active components–target network and Kyoto Encyclopedia of Genes and Genomes (KEGG) analysis. The “Degree” of each component in the network was then derived, and the top 20% of targets were selected for further analyses.

### Bioinformatical gene ontology and Kyoto encyclopedia of genes and genomes pathway enrichment analysis

We next performed the GO and KEGG analyses of the intersecting genes. The GO terms included biological processes (BPs), cellular components (CCs), and molecular functions (MFs). The GO and KEGG analyses were performed using Metascape software^8^ with the feature-rich tool. A *p-*value of <0.05 was considered statistically significant.

### Construction of the target protein–protein interaction (PPI) network

The data on the common targets of ELJN and ISHL were imported into the STRING V11.5 database^9^ (18), and the species “Homo sapiens” was selected to create the PPI network. Next, we derived these common targets using a Venn diagram, matched the drug components with the common targets, and then imported the matching results into Cytoscape (version 3.9.1) to construct the drug component and common target networks (19).

### Molecular docking analysis

We performed molecular docking analysis of the target and corresponding active components from the above procedures using the retrieved InChlKey components from the TCMSP and PubChem databases (20), in order to retrieve the corresponding ligand data as a file. After converting it using the Chem3D software (version 20.0.0), the receptor files for the corresponding targets were retrieved from the RCSB PDB database^10^ (21). The water and residue ligands of the receptors were removed using Pymol (version 2.5.0), while the receptor and ligand were converted to their secondary structures using the Autodock Tools (version 1.5.7) (22) and docked in the Autodock Vina software (version 1.1.2) (23). The molecular docking data were transferred into Pymol (version 2.5.0) for visualization.

## Results

### Identification of ELJN active components and targets

Erlongjiaonang, as in a powder form, contains 10 different TCM herbs, i.e., Gardeniae Fructus, Alisma, Caulis Akebiae, Gentianae Radix Et Rhozima, Anemone altaica Fisch, Rehmanniae Radix Praeparata, Angelicae Sinensis Radix, Licorice, Scutellariae Radix, and Antelope Horn. We then searched the constituent herbs of ELJN in the TCMSP database and reviewed the relevant PubMed literature, as well as considered Lipinski’s rules (24) and found a total of 147 active components, including 10 active components of Gentianae Radix Et Rhozima, 36 active components of Scutellariae Radix, seven active components of Alisma, 10 active components of Zedoary, eight active components of Caulis Akebiae, 15 active components of Gardeniae Fructus, two active components of Angelicae Sinensis Radix, five active components in Anemone Altaica Fisch, and 92 active components in Licorice, but there were no active components of Antelope Horn.

**Figure 2:**
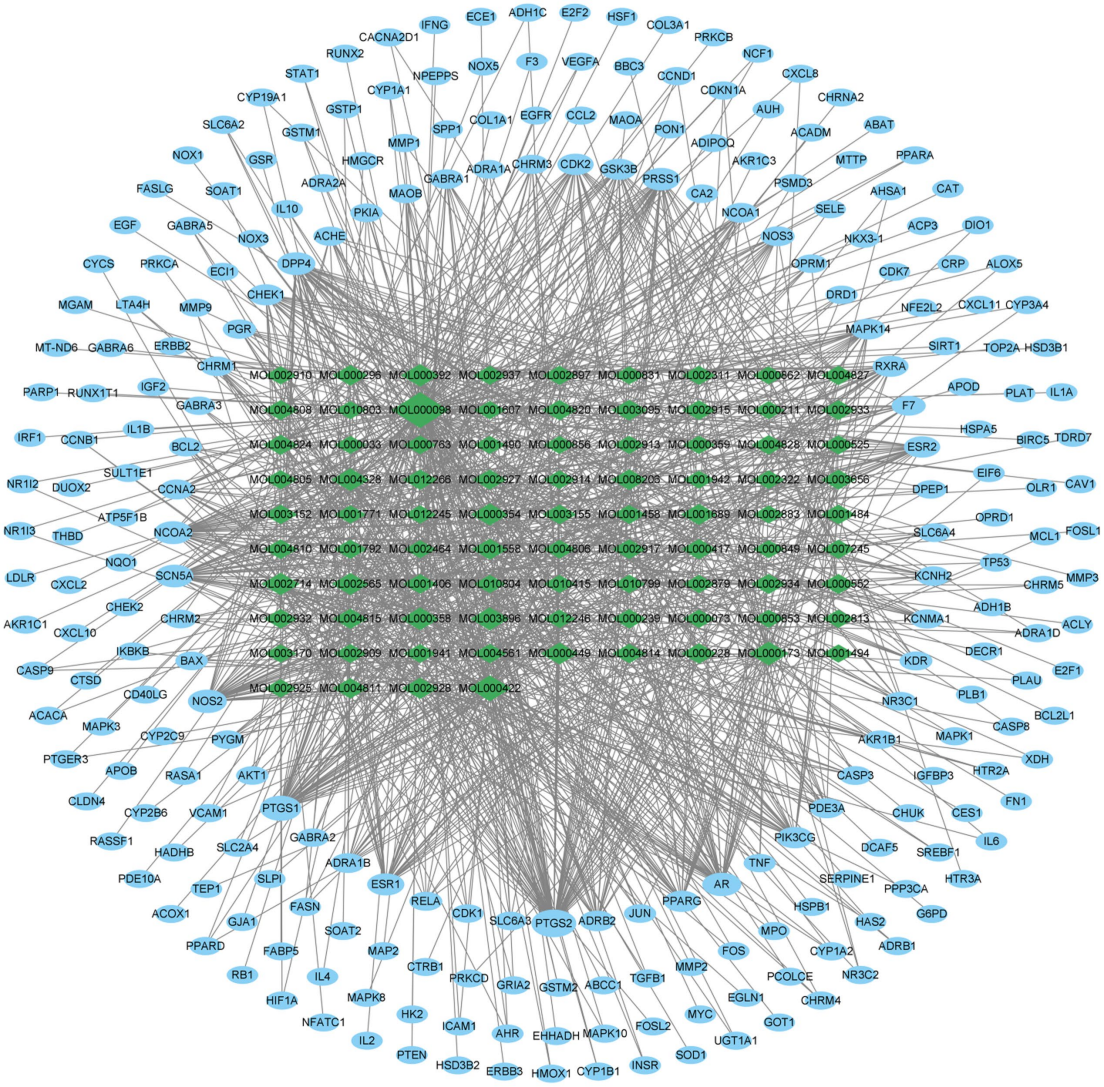
Drug components–target network. The green ovals correspond to the compositions, while the blue ovals refer to the targets. The more lines connected, the stronger the correlation.

Subsequently, we utilized the 85 active components in ELJN, using the cutoff values of >30% OB, and > 0.18 DL, and obtained 250 gene targets accordingly (Supplementary Table S1); thus, we then constructed the component–target network using Cytoscape (version 3.9.1; Figure 2). Among them, we ranked these components according to the “degree” value for the top nine components (Supplementary Table S2), while based on the “Closeness Centrality” value, we identified the top 20 gene targets of the active components in ELJN (Supplementary Table S3).

### Common targets of ELJN and ISHL and bioinformatical data

**Figure 3:**
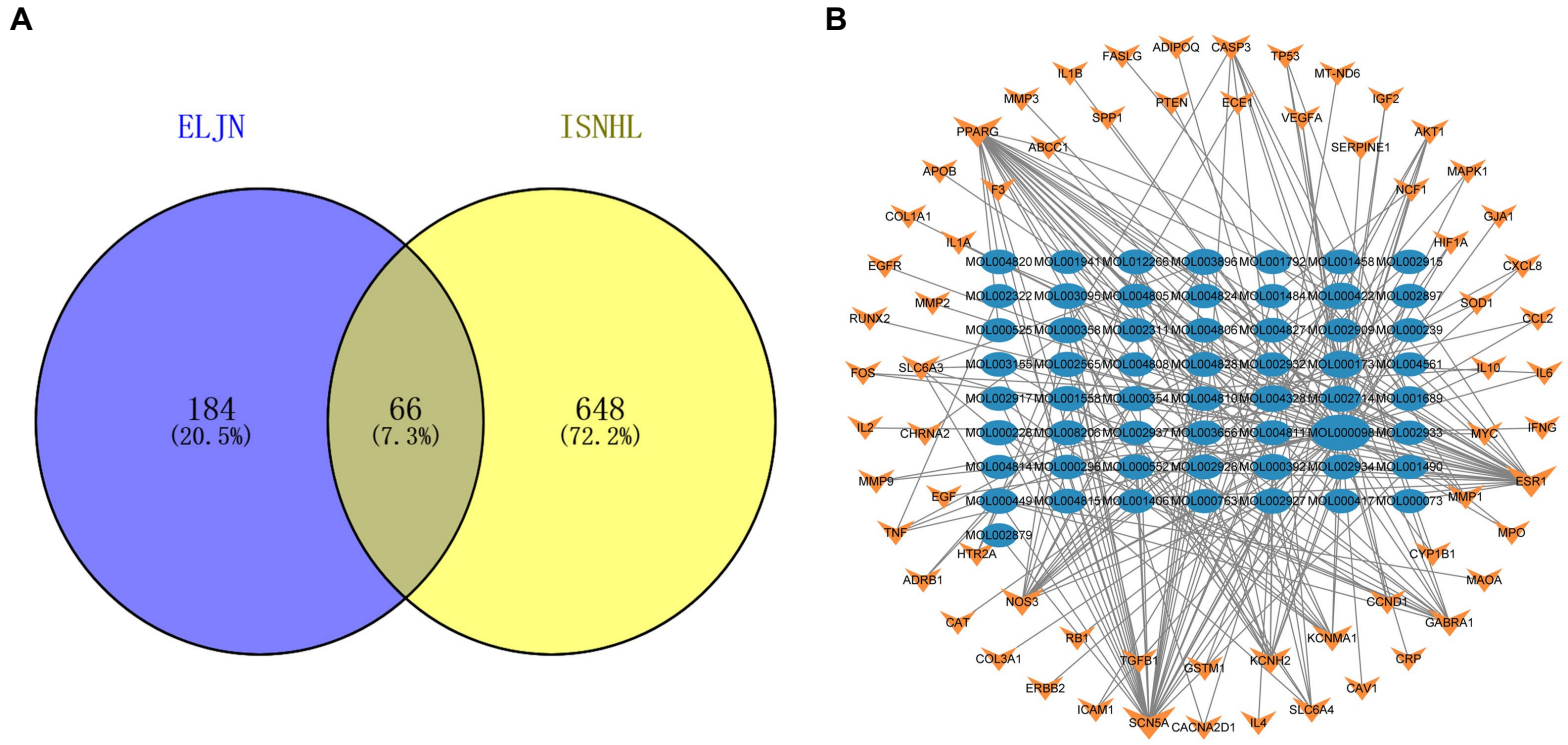
Intersection target Venn diagram and the drug components–intersection target network. **(A)** The purple corresponds to the ELJN targets, while the yellow refers to the ISHL gene targets and the gray is their intersection component. **(B)** The blue oval corresponds to the drug component, while the orange color refers to the intersection target of the drug and disease. The more lines connected, the stronger the correlation.

Similarly, we obtained a total of 714 ISHL-related gene targets after searching the OMIM, Genecards, and TTD databases. We then constructed a Venn diagram using Venn 2.1 to illustrate the connection between ELJN and ISHL with 66 drug–disease intersection targets (Figure 3A). After combining the 66 gene targets, we built the drug components–intersecting target network (Figure 3B).

**Figure 4:**
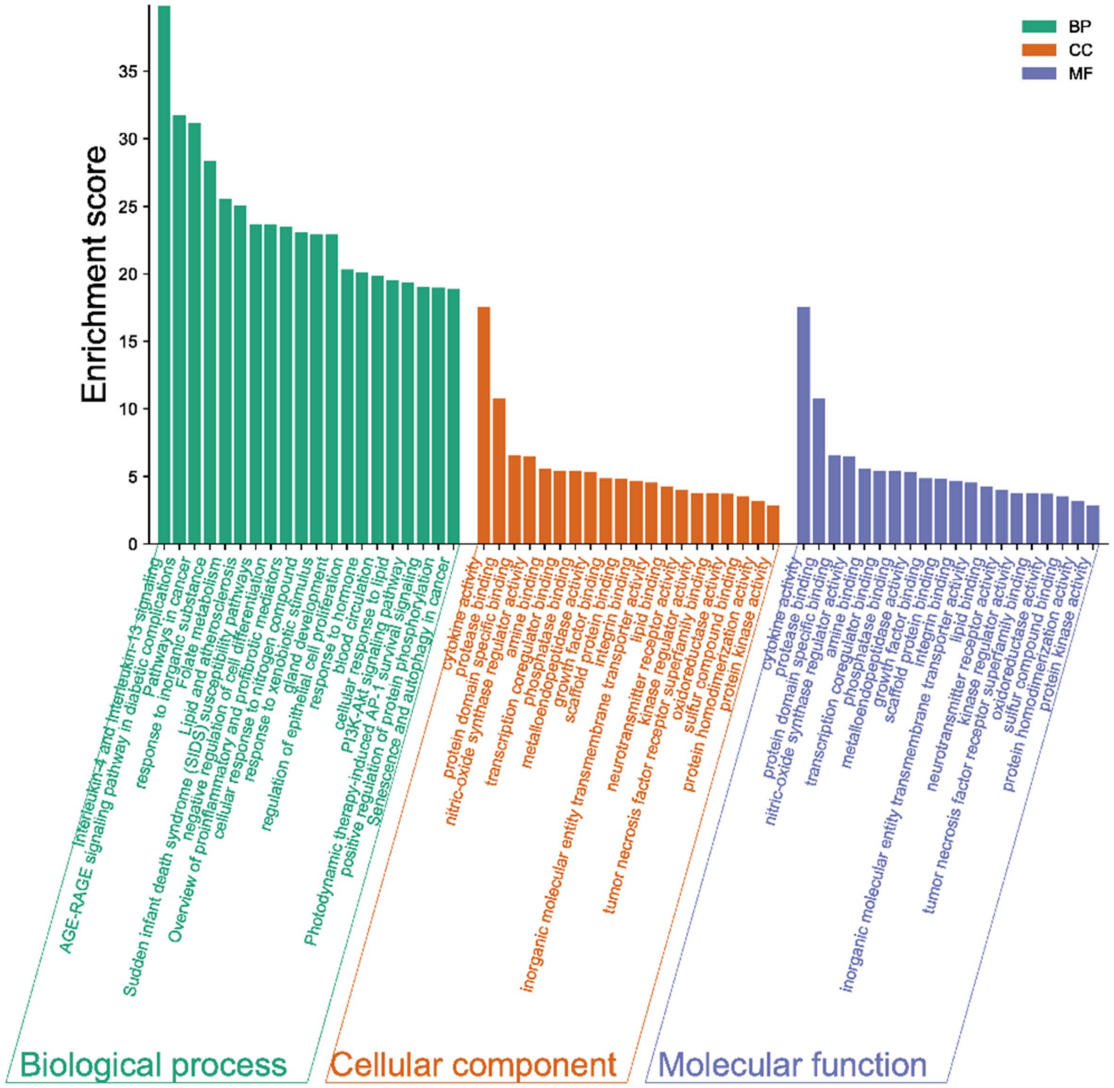
Gene ontology (GO) terms. The green bars represent the biological processes (BPs), while the orange refers to the cellular components (CCs), and the blue shows the molecular functions (MFs) of the genes. The vertical axis is the enrichment fractions.

The results of the GO analysis show that mainly enriched biological processes, such as the responses to stimuli, regulation of biological processes, multicellular organismal processes, negative regulation of biological processes, positive regulation of biological processes, metabolic processes, biological processes involved in interspecies interaction between organisms, signaling pathways, biological regulation, growth, immune system processes, and many others. Furthermore, the enriched cellular components included cytokine activity, protease binding, protein domain-specific binding, phosphatase binding, metalloendopeptidase activity, scaffold protein binding, inorganic molecular entity transmembrane transporter activity, lipid binding, neurotransmitter receptor activity, kinase regulator activity, oxidoreductase activity, protein homodimerization activity, and protein kinase activity (Figure 4).

**Figure 5:**
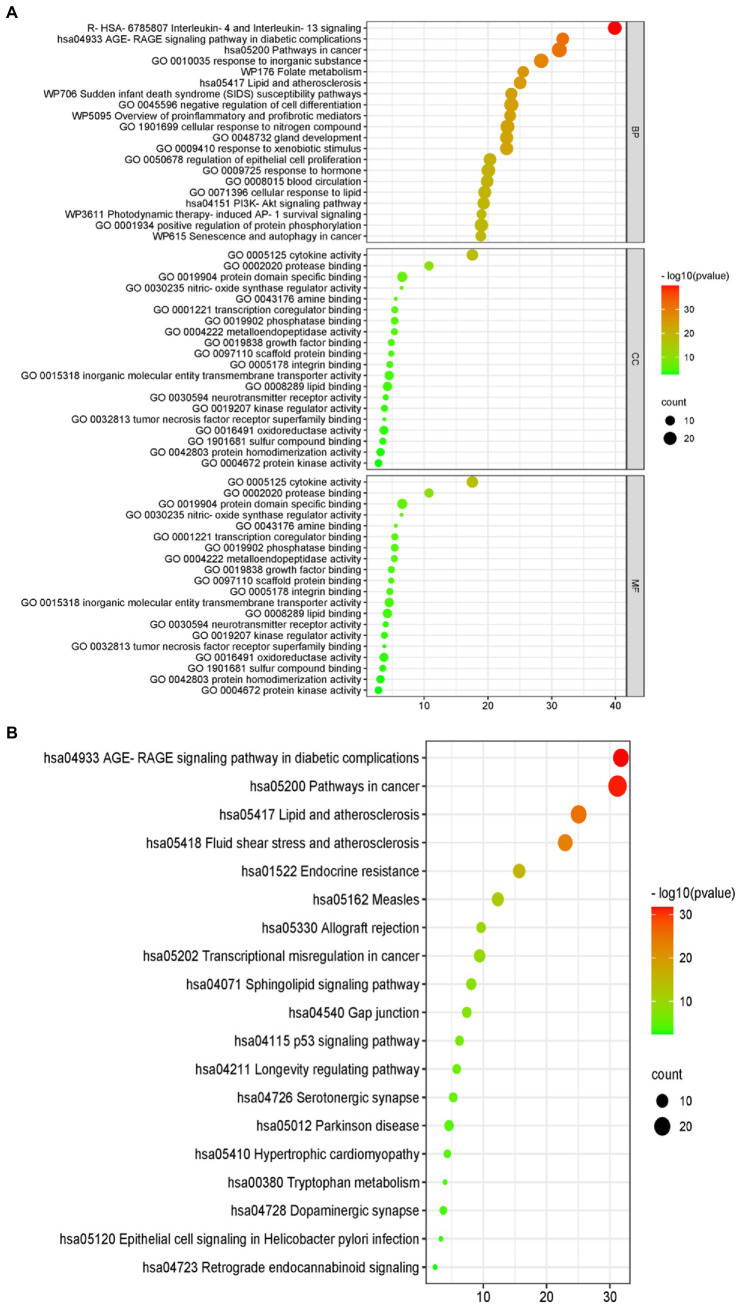
Gene ontology (GO) and the Kyoto Encyclopedia of Genes and Genomes (KEGG) analyses. **(A)** The GO analytic bubble diagram. **(B)** The KEGG analytic bubble diagram. The size of the circle represents the number of genes enriched in the pathway. The larger the circle, the greater the number of enriched proteins, and the color represents different *p*-values.

**Figure 6:**
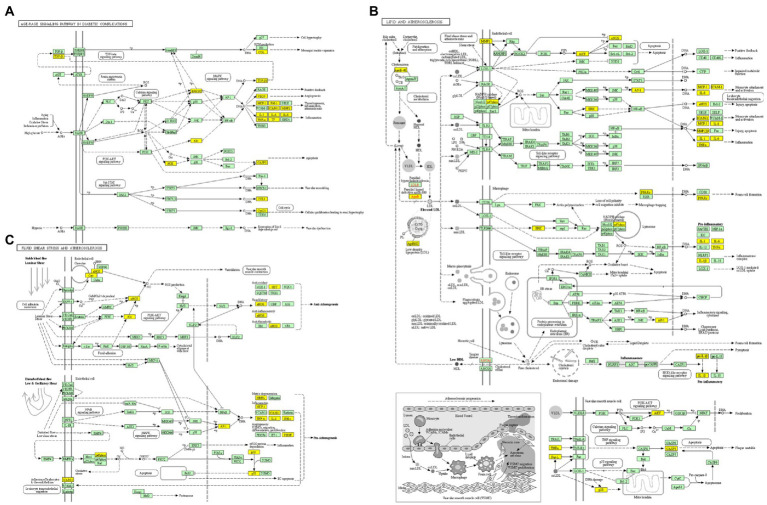
KEGG enrichment analysis pathway diagram. **(A)** The pathway map of AGE-RAGE signaling pathway in diabetic complications. **(B)** The pathway map of lipid and atherosclerosis. **(C)** The pathway map of fluid shear stress and atherosclerosis. The non-white targets in the pathway map are the intersection targets of ELJN and ISHL, while the yellow color is the possible targets present in the pathway as a result of KEGG enrichment analysis.

Furthermore, there were 157 relevant metabolic pathways after the KEGG analysis. The key KEGG enrichment pathways of ELJN anti-ISHL were the AGE-RAGE signaling pathway in diabetic complications, along with pathways in cancer, lipid and atherosclerosis, fluid shear stress and atherosclerosis, endocrine resistance, measles, allograft rejection, transcriptional misregulation in cancer, sphingolipid signaling pathway, gap junction, the p53 signaling pathway, the longevity regulating pathway, serotonergic synapses, Parkinson’s disease, hypertrophic cardiomyopathy, tryptophan metabolism, dopaminergic synapse, epithelial cell signaling in *Helicobacter pylori* infection, and retrograde endocannabinoid signaling (Figures 5, 6).

### Identification of the protein–protein interaction (PPI) and active components–drug–disease intersection target–pathway networks

**Figure 7:**
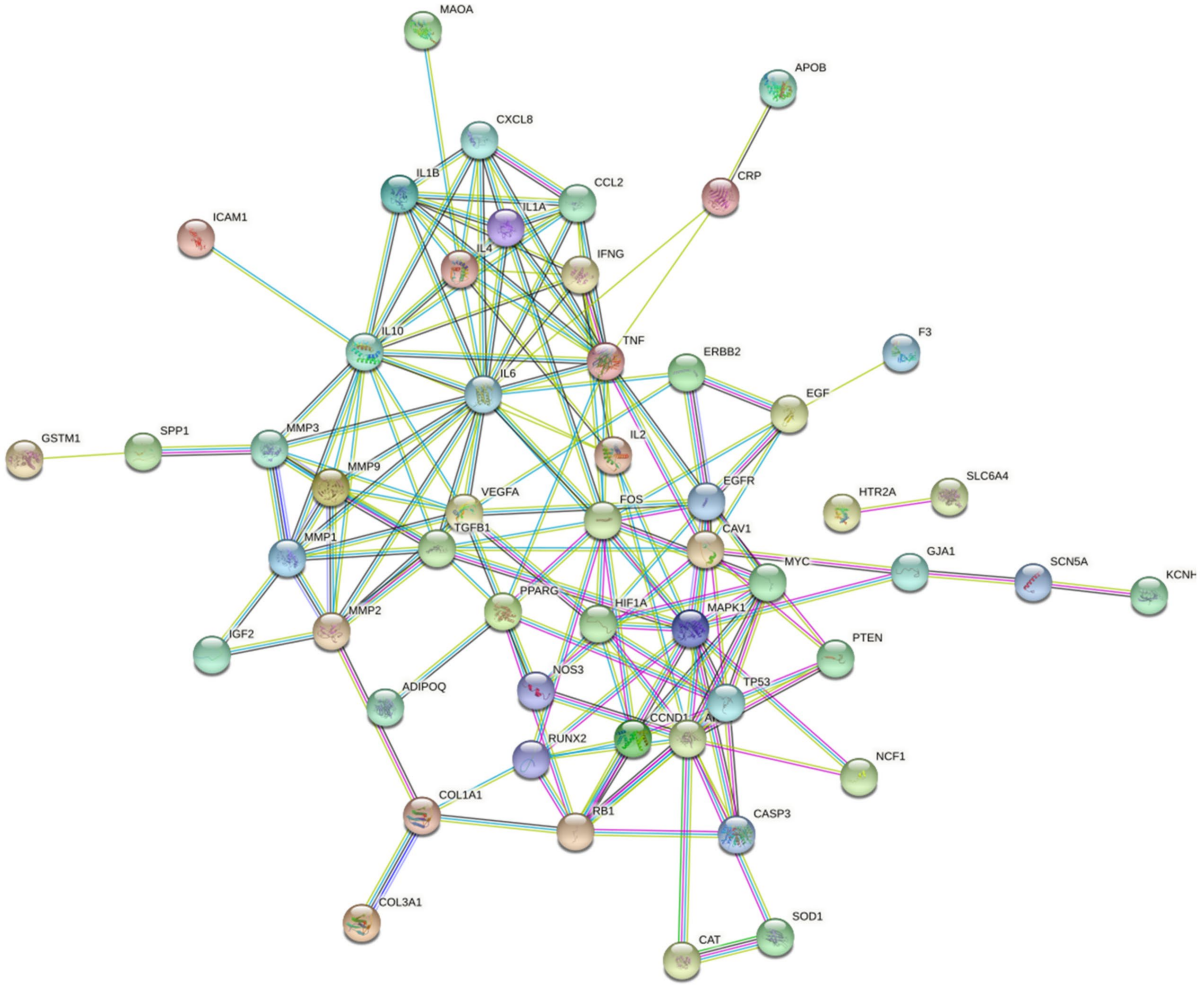
Protein–protein interaction (PPI) network. The more lines connected, the stronger the correlation.

Next, we performed a PPI network analysis (Figure 7), and SCN5A, ESR1, PPARG, NOS3, GABRA1, and KCNH2 were the six gene targets with the most connected genes. However, according to the “combined_score” value, we obtained 20 targets (Supplementary Table S4).

We then mapped the ELJN active components–intersection target–pathway network using Cytoscape (version 3.9.1). We ranked the intersection targets according to the “degree” value for the top 10 intersection gene targets (Supplementary Tables S5, S6). Next, we compared the top 20 targets of each from the component–target network and the PPI network analysis, and the top 10 targets of the components–target–signaling pathway network and found that nitric oxide synthase 3 (NOS3) and AKT1 were visible in all three data sets, suggesting a strong association of NOS3 and AKT1 with ISHL during ELJN treatment.

**Figure 8:**
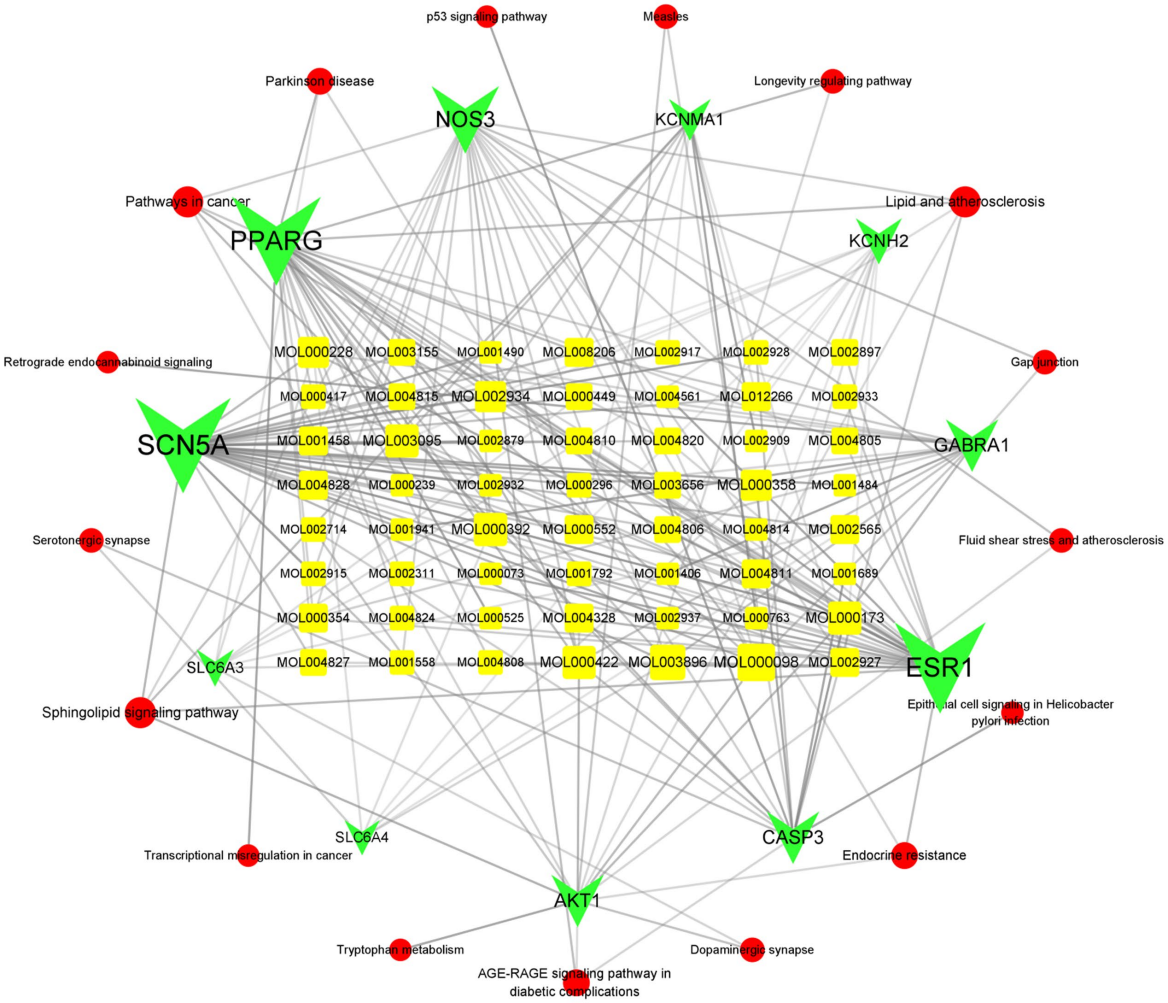
Active components–intersection target–signaling pathway network. The circular red color refers to the signal pathways, while the green color corresponds to the targets and the yellow color is the active component. The more lines connected, the stronger the correlation.

In addition, we searched for components that are associated with these two genes and identified isorhamnetin, formononetin, kaempferol, quercetin, naringenin, oroxylin A, 7-methoxy-2-methyl isoflavone, Glepidotin A, Salvigenin, Skullcapflavone II, 5,2′-dihydroxy-6,7,8, trimethoxyflavone, coptisine, epiberberine, rivularin, sesamin, 5-hydroxy-7-methoxy-2-(3,4,5, trimethoxyphenyl)chromone, beta-sitosterol, acacetin, wogonin, and baicalein. After narrowing these down, we concluded that isorhamnetin, formononetin, kaempferol, quercetin, and baicalein were somehow associated with ISHL (Figure 8).

### Molecular docking identification of ELJN components binding to NOS3 and AKT1

**Figure 9:**
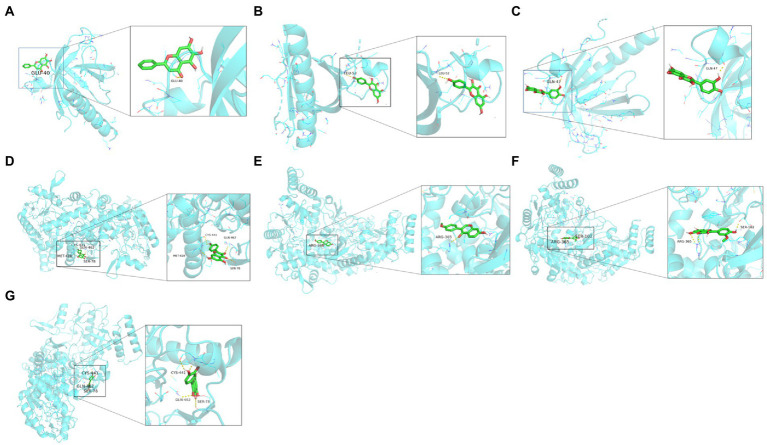
Molecular docking analytic data. **(A)** AKT1 docks with baicalein. **(B)** AKT1 docks with kaempferol. **(C)** AKT1 docks with quercetin. **(D)** NOS3 docks with kaempferol. **(E)** NOS3 docks with formononetin. **(F)** NOS3 docks with isorhamnetin. **(G)** NOS3 docks with quercetin. The blue molecular structure shows the target site, while the green molecular structure is the active component.

We then molecularly docked NOS3 and AKT1 with five ligands (isorhamnetin, formononetin, kaempferol, quercetin, and baicalein) and identified nine sets of docking results (Figure 9). The binding energies of isorhamnetin, formononetin, kaempferol, and quercetin to NOS3 were-9.8, −10.2, −9.7, and −9.9 kcal/mol, respectively, while the binding energies of kaempferol, quercetin, and baicalein to AKT1 were-6.1, −6.0, and −6.1 kcal/mol, respectively. It is generally a rule that a binding affinity below −4.5 kcal/mol indicates a weak binding capacity, while an affinity below-6 kcal/mol indicates a strong binding capacity. In our current data, this indicates that the binding between isorhamnetin, formononetin, or kaempferol vs. NOS3 or AKT1, quercetin vs. NOS3, and baicalein vs. AKT1 is stable and strong.

## Discussion

The precise etiology of ISHL remains to be defined, but multiple causes have been hypothesized, including vascular obstruction in the ear, viral infection, and/or labyrinthine membrane ruptures (25). Clinically, corticosteroids are the standard treatment course for patients with ISHL, although such a practice still has opposition as the effectiveness is relatively low (2). In China, ELJN is frequently prescribed to treat patients with ISHL by eliminating “the heat” from the liver to clear the dampness from orifices, in accordance with TCM theory (26). Indeed, the previous studies showed that ELJN had better anti-ISHL activity, especially in combination with dexamethasone than with dexamethasone alone (5), although the underlying molecular mechanism of ELJN action is unclear. Thus, our current study performed network pharmacology and molecular docking analyses to reveal the potential mechanism of ELJN in the treatment of ISHL. Our data showed that the ELJN components isorhamnetin and formononetin could target nitric oxide synthase 3 (NOS3), while the ELJN component baicalein could target AKT1, and the ELJN components kaempferol and quercetin could target both NOS3 and AKT1, as the party of the therapeutic effect of ELJN on ISHL.

Isorhamnetin, a component of ELJN, is also one of the most important active components in sea buckthorn fruit and ginkgo biloba (27). It is able to inhibit renal angiotensin-II-induced cardiac hypertrophy and fibrosis by manipulating transforming growth factor beta (TGF-β) signaling (28) or by altering the activity of the renal angiotensin system. Isorhamnetin also protected against concanavalin A-induced acute fulminant hepatitis (AFH) in a mouse model by inhibiting p38 phosphorylation and promoting PPAR-α expression, which in turn prevented the inflammatory responses and blocked cell apoptosis and autophagy in the mouse liver (29). In the current study, we identified that isorhamnetin could target NOS3, an enzyme localized in the endothelium that functions to synthesize nitric oxide (NO) for different biological activities (30), such as the promotion of vascular relaxation (31) and blood pressure control (32). Previous studies have shown that NOS3 deficiency could lead to the impairment of blood microcirculation (33). In contrast, an increase in NOS3 expression in brain ischemia–reperfusion injury improved and released neuronal injury but inhibited tissue inflammation, oxidative stress, and apoptosis (34). Thus, we speculate that isorhamnetin in ELJN could target NOS3, thereby improving blood circulation in the cochlea and improving ISHL. However, such speculation requires further experimental validation.

The ELJN component formononetin is an isoflavone derived from the legume family. It is a member of the phytoestrogen class of compounds and clinically possesses antioxidative, anti-inflammatory, neuroprotective, and blood pressure-lowering activities (35). For example, formononetin has been shown to induce Kruppel-like factor 4 expression and nuclear translocation to inhibit inflammatory responses during atherosclerosis (36). In the current study, we found that formononetin could also target NOS3 in the treatment of ISHL.

In addition, the ELJN component baicalein is the most abundant active component in Scutellaria baicalensis with neuroprotective effects (37). Baicalein can attenuate neuroinflammatory responses by inhibiting the TLR4/NF-κB pathway (38, 39). Baicalein was able to modulate FOXO3a expression to inhibit reactive oxygen species (ROS) production in cardiac hypertrophy while also activating autophagy (40). Baicalein has the ability to scavenge oxygen-free radicals to protect DNA deoxyribose residues and repair double-stranded breaks (41), as well as inhibit inflammatory responses (42). Our current data showed that baicalein could bind to AKT1 in the treatment of ISHL. The Akt family of serine–threonine protein kinases consists of three isoforms, i.e., Akt1/PKB-ɑ, Akt2/PKB-β, and Akt3/PKB-γ, to form the PI3K/Akt signaling to play an important role in the regulation of cell biology (43, 44). For example, AKT1 had a protective role in oxygen–glucose deprivation (OGD)-induced cochlear cell injury (45). High-density lipoprotein-induced increases in AKT phosphorylation protected against OGD-induced cell death (46). Therefore, we believe that baicalein exerts its role in reducing the inflammatory response by manipulating AKT1 activity, thereby protecting the auditory nerve.

Kaempferol, another active component in ELJN, is from a class of flavonoids found in a variety of vegetables and fruits, such as cauliflower, beans, tomatoes, strawberries, and grapes (47). Kaempferol possessed an inhibitory effect on the expression of oxidized low-density lipoprotein (oxLDL) and the development of atherosclerosis, as well as an inhibitory effect on macrophage activation *via* CD36 inhibition (48). Kaempferol could prevent lipid peroxidation, protect hippocampal cells from oxidative damage (49), and inhibit ROS production to protect cells against oxidative stress (50). Kaempferol exerted its anti-inflammatory effects *via* the inhibition of NF-κB, MAPK, and Akt signaling (51). Our current data indicate that kaempferol binds to NOS3 and AKT1 in the context of ELJN-induced treatment of ISHL. Another active component of ELJN, quercetin, is a flavonoid found in various plants, including onions, apples, grapes, nuts, tea, and the bark of plants (52). Quercetin possesses anti-inflammatory and neuroprotective effects and activity in treating cardiovascular disease (53–55). Our current data speculated that the ELJN anti-ISHL activity could be a result of quercetin binding to NOS3 and AKT1.

Again, we found that naringenin, oroxylin A, 7-methoxy-2-methyl isoflavone, Glepidotin A, Salvigenin, Skullcapflavone II, 5,2′-dihydroxy-6,7,8, trimethoxyflavone, coptisine, epiberberine, rivularin, sesamin, 5-hydroxy-7-methoxy-2-(3,4,5, trimethoxyphenyl)chromone, beta-sitosterol, acacetin, and wogonin might have similar effects in the therapeutic process, but we excluded them after our screening, perhaps more evidence for their effects will be available later.

In conclusion, our network pharmacology and molecular docking analyses revealed the potential gene targets of both ELJN and ISHL. The effect of ELJN treatment on ISHL could be due to the ELJN components isorhamnetin, formononetin, baicalein, kaempferol, and quercetin binding to AKT1 and NOS3 to reduce inflammatory responses and damage to inner ear hair cells. Further studies are needed to experimentally confirm these data.

## Data availbility statement

The original contributions presented in the study are included in the article/Supplementary material, further inquiries can be directed to the corresponding authors.

## Ethics statement

Ethical review and approval was not required for the study on human participants in accordance with the local legislation and institutional requirements. Written informed consent from the patients/participants or patients/participants’ legal guardian/next of kin was not required to participate in this study in accordance with the national legislation and the institutional requirements.

## Author contributions

XS, YY, and YaS: conceptualization of the study. HZ and YW: data acquisition. CX and JQ: data analysis and processing. HZ, YW, and CX: software research. GL, YuS, JQ, LC, XS, YY, and YaS: supervision and design of this study. HZ, YW, and CX: preparation of the original draft of this manuscript. XS, YY, and YaS: manuscript revision, critical review and editing. All authors contributed to the article and approved the submitted version.

## Funding

This work was supported in part by grants from Natural Science Foundation of Shandong Province (ZR2021MH378), Natural Science Foundation of Shandong Province (ZR2022QH073), Shandong Society of Geriatrics science and technology project (LKJGG2021Z020), and the Yantai Science and Technology Innovation Development Project (2021MSGY046).

## Conflict of interest

The authors declare that this research was conducted in the absence of any commercial or financial support that could be considered a conflict of interest.

## Publisher’s note

All claims expressed in this article are solely those of the authors and do not necessarily represent those of their affiliated organizations, or those of the publisher, the editors and the reviewers. Any product that may be evaluated in this article, or claim that may be made by its manufacturer, is not guaranteed or endorsed by the publisher.

## Supplementary material

The Supplementary material for this article can be found online at: https://www.frontiersin.org/articles/10.3389/fneur.2023.1121738/full#supplementary-material

## Footnotes

^1^ https://tcmspw.com/

^2^ http://www.swisstargetprediction.ch/

^3^ https://www.uniprot.org/

^4^ https://omim.org/

^5^ https://www.genecards.org/

^6^ http://db.idrblab.net/ttd/

^7^ https://bioinfogp.cnb.csic.es/tools/venny/

^8^ https://metascape.org/gp/index.html#/main/step1

^9^ https://cn.string-db.org/

^10^ https://www.rcsb.org/
