# Supplementary material for: Potential molecular mechanisms of Erlongjiaonang action in idiopathic sudden hearing loss: A network pharmacology and molecular docking analyses
Source: Front Neurol. 2023 Mar 30;14:1121738. doi: 10.3389/fneur.2023.1121738 (PMC10098218; doi:10.3389/fneur.2023.1121738)
Supplement: Supplementary file 1 [file Table_1.DOCX]

***Supplementary Material***

**Potential molecular mechanisms of Erlongjiaonang action on idiopathic sudden hearing loss: A network pharmacology and molecular docking analysis**

**He Zhao^a,b,c,^** **^†^, Yan Wang^a,b,c,^** **^†^, Yuwan Song^b,c,^** **^†^, Guangjin Li^b,c,d^ , Cong Xu^a,b,c^, Jingjing Qiu^b,c^, Limei Cui^b,c^, Xicheng Song^b, c,^*, Yujuan Yang^b,c,^*, Yan Sun^b,c,^***

^a^The Second Medical College of Binzhou Medical University，Yantai 264000,Shandong, China.

^b^Department of Otolaryngology, Head and Neck Surgery, Yantai Yuhuangding Hospital, Qingdao University；Yantai 264000, Shandong, China

^c^Shandong Provincial Clinical Research Center for Otorhinolaryngologic Diseases. Yantai 264000, Shandong, China

^d^School of Clinical Medicine, Weifang Medical University, Weifang261053, China

^†^ These three authors contribute equally to this work as the co-first authors.

* **Correspondence:**

Yan Sun

[entsunyan@126.com](mailto:entsunyan@126.com)

Xicheng Song

[drxchsong@163.com](mailto:drxchsong@163.com)

Yujuan Yang

[yangyujuanyyj@163.com](mailto:yangyujuanyyj@163.com)

**Supplementary Files**

Table S1. Molid correspondence to the component names

| molid | component |
| --- | --- |
| MOL000358 | beta-sitosterol |
| MOL000449 | Stigmasterol |
| MOL000033 | (3S,8S,9S,10R,13R,14S,17R)-10,13-dimethyl-17-[(2R,5S)-5-propan-2-yloctan-2-yl]-2,3,4,7,8,9,11,12,14,15,16,17-dodecahydro-1H-cyclopenta[a]phenanthren-3-ol |
| MOL000359 | sitosterol |
| MOL001607 | ZINC03982454 |
| MOL001771 | poriferast-5-en-3beta-ol |
| MOL002813 | Aucubin |
| MOL001484 | Inermine |
| MOL001792 | DFV |
| MOL000211 | Mairin |
| MOL002311 | Glycyrol |
| MOL000239 | Jaranol |
| MOL002565 | Medicarpin |
| MOL000354 | isorhamnetin |
| MOL003656 | Lupiwighteone |
| MOL003896 | 7-Methoxy-2-methyl isoflavone |
| MOL000392 | formononetin |
| MOL000417 | Calycosin |
| MOL000422 | kaempferol |
| MOL004328 | naringenin |
| MOL004805 | (2S)-2-[4-hydroxy-3-(3-methylbut-2-enyl)phenyl]-8,8-dimethyl-2,3-dihydropyrano[2,3-f]chromen-4-one |
| MOL004806 | euchrenone |
| MOL004808 | glyasperin B |
| MOL004810 | glyasperin F |
| MOL004811 | Glyasperin C |
| MOL004814 | Isotrifoliol |
| MOL004815 | (E)-1-(2,4-dihydroxyphenyl)-3-(2,2-dimethylchromen-6-yl)prop-2-en-1-one |
| MOL004820 | kanzonols W |
| MOL004824 | (2S)-6-(2,4-dihydroxyphenyl)-2-(2-hydroxypropan-2-yl)-4-methoxy-2,3-dihydrofuro[3,2-g]chromen-7-one |
| MOL004827 | Semilicoisoflavone B |
| MOL004828 | Glepidotin A |
| MOL001689 | acacetin |
| MOL000173 | wogonin |
| MOL000228 | (2R)-7-hydroxy-5-methoxy-2-phenylchroman-4-one |
| MOL002714 | baicalein |
| MOL002909 | 5,7,2,5-tetrahydroxy-8,6-dimethoxyflavone |
| MOL002910 | Carthamidin |
| MOL002913 | Dihydrobaicalin_qt |
| MOL002914 | Eriodyctiol (flavanone) |
| MOL002915 | Salvigenin |
| MOL002917 | 5,2',6'-Trihydroxy-7,8-dimethoxyflavone |
| MOL002925 | 5,7,2',6'-Tetrahydroxyflavone |
| MOL002927 | Skullcapflavone II |
| MOL002928 | oroxylin a |
| MOL002932 | Panicolin |
| MOL002933 | 5,7,4'-Trihydroxy-8-methoxyflavone |
| MOL002934 | NEOBAICALEIN |
| MOL002937 | DIHYDROOROXYLIN |
| MOL000525 | Norwogonin |
| MOL000552 | 5,2'-Dihydroxy-6,7,8-trimethoxyflavone |
| MOL000073 | ent-Epicatechin |
| MOL001458 | coptisine |
| MOL001490 | bis[(2S)-2-ethylhexyl] benzene-1,2-dicarboxylate |
| MOL002879 | Diop |
| MOL002897 | epiberberine |
| MOL008206 | Moslosooflavone |
| MOL010415 | 11,13-Eicosadienoic acid, methyl ester |
| MOL012245 | 5,7,4'-trihydroxy-6-methoxyflavanone |
| MOL012246 | 5,7,4'-trihydroxy-8-methoxyflavanone |
| MOL012266 | rivularin |
| MOL002322 | isovitexin |
| MOL003155 | pranferin |
| MOL001558 | sesamin |
| MOL003152 | Gentisin |
| MOL003170 | Gentisein |
| MOL010799 | Ariskanin A |
| MOL010803 | aristolochic acid A |
| MOL010804 | aristolochic acid II |
| MOL000296 | hederagenin |
| MOL000763 | Aristoloside_qt |
| MOL002464 | 1-Monolinolein |
| MOL000849 | 16β-methoxyalisol B monoacetate |
| MOL000831 | Alisol B monoacetate |
| MOL000862 | [(1S,3R)-1-[(2R)-3,3-dimethyloxiran-2-yl]-3-[(5R,8S,9S,10S,11S,14R)-11-hydroxy-4,4,8,10,14-pentamethyl-3-oxo-1,2,5,6,7,9,11,12,15,16-decahydrocyclopenta[a]phenanthren-17-yl]butyl] acetate |
| MOL000853 | alisol B |
| MOL000856 | alisol C monoacetate |
| MOL001406 | crocetin |
| MOL001941 | Ammidin |
| MOL004561 | Sudan III |
| MOL000098 | quercetin |
| MOL001494 | Mandenol |
| MOL001942 | isoimperatorin |
| MOL002883 | Ethyl oleate (NF) |
| MOL003095 | 5-hydroxy-7-methoxy-2-(3,4,5-trimethoxyphenyl)chromone |
| MOL007245 | 3-Methylkempferol |

Table S2. Table of degree of Component-Target Network Diagram.

We derived the results based on the plotted component-target networks and obtained the components of them with a degree ≥ 30.

| MOL name | Gene name | Degree |
| --- | --- | --- |
| MOL000098 | quercetin | 130 |
| MOL000422 | kaempferol | 57 |
| MOL000173 | wogonin | 42 |
| MOL003896 | 7-Methoxy-2-methyl isoflavone | 36 |
| MOL000358 | beta-sitosterol | 34 |
| MOL000392 | formononetin | 34 |
| MOL004328 | naringenin | 34 |
| MOL002714 | baicalein | 33 |
| MOL000354 | isorhamnetin | 30 |

Table S3. Table of Closeness Centrality of Component-Target Network Diagram.

We derived the results based on the plotted component-target network, and the top 20 targets were obtained based on their Closeness Centrality ranking.

| name | Closeness Centrality |
| --- | --- |
| PTGS2 | 0.543089 |
| PTGS1 | 0.4919 |
| NCOA2 | 0.430968 |
| PIK3CG | 0.429858 |
| SCN5A | 0.426564 |
| AR | 0.425478 |
| DPP4 | 0.423321 |
| PRSS1 | 0.423321 |
| PPARG | 0.413879 |
| RXRA | 0.406821 |
| ADRB2 | 0.4 |
| BCL2 | 0.399044 |
| NOS3 | 0.393404 |
| GABRA1 | 0.389732 |
| RELA | 0.389732 |
| AKT1 | 0.380844 |
| BAX | 0.37655 |
| JUN | 0.37402 |
| ACHE | 0.372352 |
| MAOB | 0.369878 |

Table S4. Table of PPI network diagram results.

We exported the results in the PPI network diagram, based on the "combined_score" > 0.995, and obtained 20 targets after de-weighting the results.

| #node1 | node2 | combined_score |
| --- | --- | --- |
| AKT1 | NOS3 | 0.999 |
| CAV1 | EGFR | 0.999 |
| CAV1 | NOS3 | 0.999 |
| EGF | ERBB2 | 0.999 |
| EGF | EGFR | 0.999 |
| EGFR | EGF | 0.999 |
| EGFR | CAV1 | 0.999 |
| ERBB2 | EGF | 0.999 |
| HIF1A | TP53 | 0.999 |
| NOS3 | CAV1 | 0.999 |
| NOS3 | AKT1 | 0.999 |
| PTEN | TP53 | 0.999 |
| TP53 | HIF1A | 0.999 |
| TP53 | PTEN | 0.999 |
| CCND1 | RB1 | 0.998 |
| CXCL8 | IL1B | 0.998 |
| EGFR | ERBB2 | 0.998 |
| ERBB2 | EGFR | 0.998 |
| HIF1A | MYC | 0.998 |
| HIF1A | VEGFA | 0.998 |
| IL1B | CXCL8 | 0.998 |
| MYC | HIF1A | 0.998 |
| RB1 | CCND1 | 0.998 |
| VEGFA | HIF1A | 0.998 |
| CXCL8 | IL6 | 0.997 |
| IL1B | IL6 | 0.997 |
| IL6 | IL1B | 0.997 |
| IL6 | CXCL8 | 0.997 |
| CAT | SOD1 | 0.996 |
| IL10 | TNF | 0.996 |
| SOD1 | CAT | 0.996 |
| TNF | IL10 | 0.996 |

Table S5.The KEGG enrichment analysis of pathways and the corresponding genes of each pathway

| Pathway | Genes |
| --- | --- |
| AGE-RAGE signaling pathway in diabetic complications | AKT1,CCND1,CASP3,COL1A1,COL3A1,F3,ICAM1,IL1A,IL1B,IL6,CXCL8,MMP2,NOS3,SERPINE1,MAPK1,CCL2,TGFB1,TNF,VEGFA,EGFR,FOS,MMP1,MMP9,ND6,PTEN,NCF1 |
| Pathways in cancer | AKT1,FASLG,CCND1,CASP3,EGF,EGFR,ERBB2,ESR1,FOS,GSTM1,HIF1A,IFNG,IGF2,IL2,IL4,IL6,CXCL8,MMP1,MMP2,MMP9,MYC,PPARG,MAPK1,PTEN,RB1,TGFB1,TP53,VEGFA,CAV1,COL1A1,TNF,NOS3,SPP1,CACNA2D1,IL1A,IL1B,CCL2,CAT,CYP1B1,ND6,SOD1,NCF1,MMP3,ICAM1,SERPINE1,ADRB1,IL10,ABCC1,MPO |
| Lipid and atherosclerosis | AKT1,APOB,FASLG,CASP3,FOS,ICAM1,IL1B,IL6,CXCL8,MMP1,MMP3,MMP9,NOS3,PPARG,MAPK1,CCL2,TNF,TP53,NCF1,IFNG,IL2,IL10,SERPINE1,TGFB1,IL1A,VEGFA,IL4,ADIPOQ,COL1A1,COL3A1,HIF1A,EGFR,CCND1,SPP1,CAV1,ND6,SOD1,MYC,CAT,SLC6A3,ABCC1 |
| Fluid shear stress and atherosclerosis | AKT1,CAV1,FOS,GSTM1,ICAM1,IFNG,IL1A,IL1B,MMP2,MMP9,NOS3,CCL2,TNF,TP53,VEGFA,NCF1 |
| Endocrine resistance | AKT1,CCND1,EGFR,ERBB2,ESR1,FOS,MMP2,MMP9,MAPK1,RB1,TP53,NOS3,CACNA2D1,RUNX2 |
| Measles | AKT1,FASLG,CCND1,CASP3,FOS,IL1A,IL1B,IL2,IL6,TP53,ERBB2,GSTM1,MAPK1,TNF,ICAM1,IFNG |
| Allograft rejection | FASLG,IFNG,IL2,IL4,IL10,TNF,IL6,TGFB1 |
| Transcriptional misregulation in cancer | RUNX2,IL6,CXCL8,MMP3,MMP9,MPO,MYC,PPARG,TP53 |
| Sphingolipid signaling pathway | AKT1,ABCC1,NOS3,MAPK1,PTEN,TNF,TP53,HIF1A,TGFB1,VEGFA,CCND1,ESR1,FOS,SERPINE1,SPP1,IL6,COL1A1,COL3A1,IL4,ADRB1,CACNA2D1,SCN5A,KCNMA1,CXCL8,CCL2,NCF1,ADIPOQ,MPO |
| Gap junction | ADRB1,EGF,EGFR,GJA1,HTR2A,MAPK1,ERBB2,NOS3,VEGFA,CHRNA2,GABRA1 |
| p53 signaling pathway | CCND1,CASP3,SERPINE1,PTEN,TP53,CAV1,ICAM1,MYC,TGFB1 |
| Longevity regulating pathway | AKT1,CAT,PPARG,TP53,ADIPOQ,CCND1,MMP1 |
| Serotonergic synapse | CASP3,HTR2A,MAOA,MAPK1,SLC6A4 |
| Parkinson disease | CASP3,MAOA,ND6,SLC6A3,SOD1,TP53,CAT,TNF,PPARG |
| Hypertrophic cardiomyopathy | CACNA2D1,IL6,TGFB1,TNF,ADRB1 |
| Tryptophan metabolism | CAT,CYP1B1,MAOA,AKT1,SOD1 |
| Dopaminergic synapse | AKT1,FOS,MAOA,SLC6A3,MAPK1 |
| Epithelial cell signaling in Helicobacter pylori infection | CASP3,EGFR,CXCL8 |
| Retrograde endocannabinoid signaling | GABRA1,ND6,MAPK1 |

Table S6. Table of degree of ELJN active components-intersection target-pathway network.

We derived the results based on the ELJN active components-intersection target-pathway network, and in the results we ranked the targets in descending order based on the degree, resulting in the top ten targets.

| Gene name | Degree |
| --- | --- |
| SCN5A | 35 |
| ESR1 | 33 |
| PPARG | 32 |
| NOS3 | 22 |
| CASP3 | 15 |
| AKT1 | 15 |
| GABRA1 | 15 |
| KCNH2 | 12 |
| KCNMA1 | 10 |
| SLC6A3 | 8 |
